# Assessment of knowledge, attitude and practices among dental practitioners on methods of infection control while carrying out dental procedures during novel coronavirus (COVID-19) pandemic

**DOI:** 10.11604/pamj.2021.39.265.29003

**Published:** 2021-08-24

**Authors:** Neetha Shenoy, Vasudev Ballal, Usha Rani, Himani Kotian, Vani Lakshmi

**Affiliations:** 1 Department of Conservative Dentistry and Endodontics, Manipal College of Dental Sciences, Manipal Academy of Higher Education, Manipal, India,; 2 Department of Health Innovation, Prasanna School of Public Health, Manipal Academy of Higher Education, Manipal, India,; 3 Department of Community Medicine, Kasturba Medical College, Manipal Academy of Higher Education Mangalore, Manipal, India,; 4 Department of Data Science, Prasanna School of Public Health, Manipal Academy of Higher Education, Manipal, India

**Keywords:** Infection control, COVID-19 pandemic, dentists, knowledge, practice

## Abstract

**Introduction:**

dental procedures produce bio-aerosols that can carry the highly contagious COVID-19 virus. Hence, the entire dental care team has to follow the current COVID-19 related infection control protocols. The study aims to assess the knowledge, attitude, and practices associated with infection control methods during dental procedures among dental practitioners during the COVID-19 pandemic in India.

**Methods:**

the online questionnaire consisted of four sections namely, demographic details, eight questions on knowledge, four questions on attitude and eight questions on the practice of dentists during COVID-19 pandemic. One point was attributed for a correct response and zero point for an incorrect response. The study used descriptive statistics and binary logistic regression models in Jamovi 1.8.1 to establish relationships between knowledge, attitude, and practices among dental professionals and their demographic characteristics.

**Results:**

among 384 dentists, 294 (76.6%) were aware of the hand hygiene methods, 372 dentists (96.9%) were aware of the Personal Protective Equipment (PPE), 343 dentists (89.3%) recorded the body temperature of the patients. One hundred and thirty eight (138) dentists (35.9%) did not use rubber dam and 158 dentists (41.1%) were not aware of the four-handed dentistry. Among the study participants, 57.8% displayed adequate knowledge, 63.8% possessed good attitude and 93.5% followed good practices on COVID-19 appropriate infection control measures during dental procedures. The mean knowledge, attitude, and practice scores were found to be 6.61, 2.04, and 3.38 respectively. Based on multivariable binary logistic regression analysis, it is observed that males (aOR: 0.55, 95% CI 0.35-0.87; p=0.011) have a lower likelihood of having a good knowledge when compared with females after adjusting for the other independent variables in the model. Also, individuals with higher qualifications (aOR: 0.57, 95% CI 0.36-0.92; p=0.022) appear to have lower likelihood of having good knowledge on COVID-19 infection control methods during dental procedure.

**Conclusion:**

the study concludes that participants possess sound knowledge, attitude and practice on hand hygiene, PPE, patient triage and waiting area modifications at the workplace. However, poor response was noted on the use of rubber dam, remote telephonic screening and four-handed dentistry practice.

## Introduction

The epidemic of novel coronavirus disease (COVID-19), caused by Coronavirus, has become a major public health challenge globally [1]. This epidemic began in Wuhan, China, in December 2019. The World Health Organization (WHO) declared a global public health emergency against this disease in January 2020 [2]. The single-stranded Coronaviruses belong to the family Coronaviridae. The main symptoms include fever, cough (especially dry), sore throat, shortness of breath, myalgia, fatigue, and less common symptoms include sputum, headache, haemoptysis, and diarrhoea. This rapidly spreading infection typically transmits through respiratory droplets or contact with an infected person [3, 4].

This pandemic has posed an enormous challenge to healthcare professionals across the globe. It had a profound effect on dentistry primarily because dental procedures often result in the generation of aerosol particles contaminated with blood and saliva particles [5]. According to Kutter *et al*. [6], aerosols can travel more than six feet. Studies have reported that the SARS-CoV-2 virus could be detected in aerosols up to three hours postoperatively and can persist on surfaces for extended periods [7, 8]. This poses a potential risk to dental health professionals and patients. Therefore, dental professionals must be aware of proper guidelines in the time of this global emergency [9, 10]. This study aims to assess the knowledge, attitudes, and practices (KAP) on infection control measures among dental practitioners during the COVID-19 pandemic in India.

## Methods

**Study design and setting**: the present cross-sectional questionnaire-based study was carried out during October- November 2020. The study followed the Strengthening the Reporting of Observational Studies in Epidemiology (STROBE) guidelines [11]. The questionnaire was administered to dental professionals across India through Google forms which were circulated through social media platforms such as Facebook and WhatsApp.

**Study population**: the study population involves dental health professionals who are registered and active members of the Dental Council of India. Based on 5% significance level with p=0.5, the study required responses from 384 participants selected randomly from the study population.

**Data collection**: the data for the study was obtained through an online questionnaire which consisted of following sections: The first section addressed the demographic details such as age, gender, state, district, highest qualification, designation, and years of practice. The second section included eight knowledge-related questions on COVID-19 disease. The third section included four questions to ascertain the attitude about the dental practice during the pandemic and the final section comprised of eight questions to assess the practice modifications directed to prevent the spread of COVID-19. Out of the eight questions, the first four questions were considered for scoring purposes, and the remaining four questions collected responses from the dentists about their practice modifications. A scoring system was used to analyse the responses to the questions on knowledge, attitude and practice. One point was attributed for a correct response and zero points for an incorrect response. The scores obtained from the study were categorised into 'Poor' and 'Good' by using the median split method.

**Statistical analysis**: the statistical analysis for the study was carried out using Microsoft Excel 2016 and Jamovi 1.8 (a graphical user interface for CRAN R Programming) [12, 13]. The significance level was set at 5%. Descriptive statistics using frequencies, measures of central tendency (arithmetic mean) and dispersion (standard deviation) were computed for the demographic characteristics associated with the respondents and their KAP scores. Independent samples t-test, one-way Analysis of Variance (ANOVA), Chi-square test were used to compare KAP scores of different participants according to the demographic characteristics. Binary logistic regression was used to quantify the associations between demographic characteristics and KAP levels. Based on the purposeful variable selection method suggested by Bursac *et al*. [14], variables with p<0.25 in the univariate logistic regression model were incorporated into the multivariate logistic regression model.

**Ethical considerations**: this study was registered under Clinical Trials Registry India (CTRI/2020/10/028295) and was conducted after obtaining the institutional ethics committee approval (IEC: 496/2020). The participants were initially briefed about the study, and informed consent was taken before filling out the online survey form. The confidentiality of data was assured to all the participants.

## Results

**Sociodemographic characteristics and KAP scores**: Table 1 depicts the sociodemographic characteristics of the dentists who participated in this survey. Among 384 participants, 121 (31.5%) were males and 263 (68.5%) were females. The participants belonged to the age group 24-48 years with an average of 33±4.8 years. 357 (93.0%) participants belonged to the clinical specialty, and 27 (7.0%) participants belonged to the pre-clinical specialty. The knowledge scores of the participants significantly varied across education levels (p=0.032). The attitude scores of the participants significantly varied across gender (p<0.001), education levels (p=0.004), staff category (p=0.019) and years of practice (p<0.001). The practice scores of the participants significantly varied across years of practice (p<0.001).

**Table 1:** demographic variables of the participants with their knowledge, attitude and practice scores

Variable	Categories	Knowledge				Attitude			Practice		
		N(%)	Mean±SD	t/F	p-value	Mean±SD	t/F	p-value	Mean±SD	t/F	p-value
**Gender**	Female	263(68.5)	6.662±0.845	1.894	0.059	1.905±0.958	-	<0.001*	3.361±0.626	-	0.372
	Male	121(31.5)	6.479±0.941			2.322±0.959	3.963		3.421±0.588	0.893	
**Highest qualification**	BDS	170 (44.3)	6.712±0.860	2.148	0.032*	1.876±0.924	-	0.004*	3.353±0.600	-	0.439
	MDS and Above	214(55.7)	6.519±0.887			2.164±1.001	2.888		3.402±0.626	0.775	
**Staff category**	Clinician	154(40.1)	6.610±0.986	0.045	0.956	2.130±0.982	4.054	0.019*	3.338±0.659	0.585	0.558
	Academician	54(14.1)	6.630±1.033			1.815±0.585			3.407±0.533		
	Both	176(45.8)	6.591±0.719			2.023±1.058			3.409±0.598		
**Years of Practice**	Upto 5 years	261 (68.0)	6.628±0.866	0.785	0.433	1.893±0.922	-	<0.001*	3.295±0.633	-	<0.001*
	More than 5 years	123(32.0)	6.553±0.907			2.341±1.023	4.295		3.561±0.530	4.037	
**Specialty**	Clinical	357(93.0)	6.583±0.869	-	0.081	2.045±0.982	0.609	0.543	3.381±0.614	0.086	0.931
	Pre-Clinical	27(7.0)	6.889±0.974	1.751		1.926±0.917			3.370±0.629		

* denotes statistically significant at p<0.05

**Dentists' knowledge of COVID-19**: overall the mean knowledge score of the participants was 6.61±0.88 (Table 2). The majority of the participants (57.8%) possessed adequate knowledge on the mode of transmission of COVID-19, symptoms of COVID-19, hand hygiene measures, and PPE. At the same time, inadequate knowledge was observed regarding the safety of usage of ultrasonic instruments, usage of the rubber dam, and four-handed dentistry (Table 3).

**Table 2:** descriptive statistics of knowledge, attitude and practice scores

Variable	Knowledge Score	Attitude Score	Practice Score
**Mean±SD**	6.61±0.88	2.04±0.98	3.38±0.61
**Median**	7	2	3
**Minimum**	4	1	1
**Maximum**	8	4	4

**Table 3:** frequency distribution of questions pertaining to knowledge, attitude and practice Knowledge related questions

Question	Correct Answer N (%)	Incorrect Answer N (%)
1. Which of the following describes the symptoms associated with COVID-19?	336(87.5%)	48(12.5%)
2. Mode of transmission of COVID-19 virus is through respiratory droplets.	381(99.2%)	03(0.8%)
3. Rubber dam isolation is recommended for every patient.	246(64.1%)	138(35.9%)
4.Four handed dentistry is highly recommended for controlling the spread of COVID-19.	226(58.9%)	158(41.1%)
5. Ultrasonic devices can be safely used in dental office for patients.	218(56.8%)	166(43.2%)
6. Which of the following hand hygiene actions prevents transmission of the virus to the health worker?	376(97.9%)	8(2.1%)
7. The preferred method of hand hygiene for visibly soiled hands is	294 (76.6%)	90(23.4%)
8. What PPE should be worn by the dental health care providers	372(96.9%)	12(3.1%)
**Attitude related questions**		
Question	Yes N(%)	No N(%)
1. Are you scared of getting infected with COVID − 19 from a patient or a co -worker?	290 (75.5%)	94(24.5%)
2. Are you keen to start your practice in this time of pandemic?	209(54.4%)	175(45.6%)
3. Do you feel anxious about contracting COVID-19 in spite of wearing PPE?	280(72.9%)	104(27.1%)
4. Do you feel it is important to create awareness in the public regarding the prevention of the spread of COVID-19?	384(100%)	0(0%)
**Practice related questions**		
Questions	Correct Answer N(%)	Incorrect Answer N (%)
1. Do you recommend initial remote screening via telephone to identify patients with suspected or possible COVID-19 infection, prior to dental visit?	203(52.9%)	181(47.1%)
2. If a patient is coughing or sneezing, would you recommend COVID-19 clearance?	381(99.2%)	3(0.8%)
3. Are you or your staff members taking the body temperature for every patient before performing dental service?	343(89.3%)	41(10.7%)
4. Are you currently asking every patient these questions prior to treatment?	368 (95.8%)	16 (4.2%)

**Dentists' attitude towards COVID-19 appropriate behaviour**: the mean attitude score of the participants was 2.04±0.98 (Table 2). Interestingly, the majority of the participants (63.8%) showed a positive attitude towards COVID-19 appropriate behaviour at work. Though the majority of the participants (75.50%) were anxious about contracting COVID-19 during a dental procedure, a good response was observed in terms of resuming dental practice after lockdown and the need to create public awareness regarding preventing the spread of COVID-19 (Table 3).

**Figure 1:**
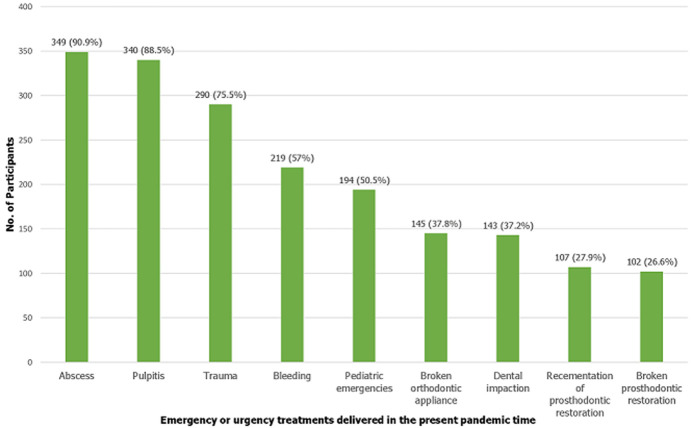
response to the question on which one of the following emergencies/urgencies are being delivered in this period in your practice?

**Figure 2:**
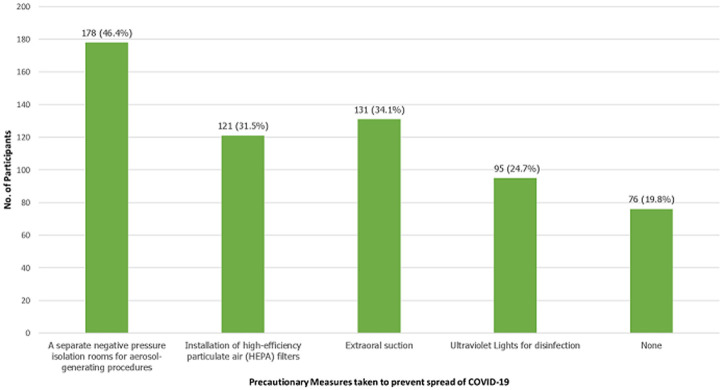
response to the question on which of the following precautionary measure have you undertaken to prevent the spread of COVID-19?

**Dentists' practice during COVID-19**: the mean practice score of the participants was 3.38±0.61 (Table 2). The majority of the dentists (93.5%) displayed good practice on various aspects, including recording body temperature, travel history, and relevant medical history regarding respiratory illness such as cough, sputum, fever, difficulty in breathing. Fifty two percent (52.9%) of dentists practiced initial telephone screening of the patients before the dental visit (Table 3). The majority of the dentists (73.4%) undertook only emergencies and urgent dental care procedures. The emergencies handled by the dentists during the COVID-19 pandemic period are outlined in Figure 1. The majority of the dentists displayed good practice at their clinic through the display of educational posters on COVID-19 (74.50%), provision for social distancing (89.3%) and a re-organised waiting area to avoid the clutter of patients (76.30%). Figure 2 shows the precautionary measures undertaken by the dentists to prevent the spread of COVID-19.

**Factors significantly associated with COVID-19 inadequate knowledge, attitude, and practice**: binary logistic regression analysis served to establish associations between the demographic characteristics and the KAP levels of the participants. Towards this, univariate binary logistic regression analysis determined the association between each demographic characteristic and knowledge, attitude, and practice levels. Further, using the purposeful variable selection method, appropriate variables were identified to carry out a multivariable binary logistic regression model (Table 4). It is observed that male (aOR: 0.55, 95% CI [0.35-0.87]; p=0.011) participants and participants with higher qualification levels (aOR: 0.57, 95% CI [0.36-0.92]; p=0.022) have a lower likelihood of having a good knowledge when compared with females. However, male dentists (aOR: 2.57, 95% CI [1.53-4.32]; p<0.001) possessed a higher likelihood of having a good COVID-19 appropriate attitude when compared with female dentists. Further, dentists with experience of more than five years [aOR: 5.85, 95% CI 1.36-25.20; p=0.018]) exhibited a higher likelihood of having good COVID-19 appropriate practices at work when compared with dentists with less than five years of experience after adjusting for the other independent variables in the model.

**Table 4:** results of binary logistic regression analysis

Variable	Levels(* indicates Reference Level)	Knowledge				Attitude				Practice			
		Univariable analysis		Multivariable Analysis		Univariable Analysis		Multivariable Analysis					
		Crude Odds Ratio (95% CI)	p-value	Adjusted Odds Ratio (95% CI)	p- value	Crude Odds Ratio (95% CI)	p-value	Adjusted Odds Ratio (95% CI)	p-value	Crude Odds Ratio (95% CI)	p-value	Adjusted Odds	p-value
Gender	Male- Female*	0.50(0.32-0.78)	0.002*	0.55(0.35-0.87)	0.011*	2.94(1.78-4.86)	<0.001*	2.57(1.53-4.32)	<0.001*	1.49(0.58- 3.84)	0.406		
Highest qualification	MDS and Above-BDS*	0.50(0.33-0.76)	0.001*	0.57(0.36-0.92)	0.022*	1.61 (1.06- 2.45)	0.026*	1.23 (0.76- 1.98)	0.401	0.99 (0.44-2.24)	0.978		
Staff Category	Clinician-Academician*	0.74(0.39- 1.41)	0.363			0.82 (0.42, 1.64)	0.580			0.16 (0.02- 1.26)	0.082		
Years of Practice	More than 5 years-Upto 5 years*	0.54(0.36-0.86)	0.008*	0.86(0.52-1.44)	0.569	2.09 (1.30, 3.37)	0.002*	1.49 (0.86- 2.59)	0.154	5.85(1.36- 25.20)	0.018*	5.85 (1.36- 25.20)	0.018*
Specialty	Clinical-Pre-Clinical*	0.56 (0.24- 1.30)	0.176**	0.49(0.20-1.18)	0.112	1.45 (0.66, 3.19)	0.357			1.16 (0.26-5.21)	0.845		

Significant at 5%, **: included for further analysis as per purposeful selection method. Level of Significance: α=5%,

## Discussion

To prevent the spread of potentially hazardous COVID-19, each dental professional has to follow strict infection control measures in addition to the ones that are routinely being followed [15, 16]. In this context, the present study has given insights into the knowledge, attitude, and practices of dental health professionals across India about infection control practices during this global pandemic. The present study observed that more than half of the participants (57.8%) had adequate knowledge, 63.8% of the participants possessed a good attitude, and 93.5% of the participants were aware of and implemented COVID-19 appropriate measures at the dental clinic. The result is comparable with the multinational survey conducted by Kamate *et al*. [10]. The good KAP scores observed in the present study could be due to the continuous dissemination of updates on infection control measures during the different unlock phases after the nationwide lockdown of 2020 [17, 18].

Recent guidelines have recommended using rubber dam and a high volume evacuator to minimise the aerosols to prevent the spread of COVID-19 [19, 20]. In the present study, few participants (35.9%) did not use the rubber dam (Table 3). Lack of training and time-consuming steps are reasons for not using rubber dams in dental practice [20, 21]. The use of a rubber dam has to be encouraged since it improves visualisation, moisture control, prevents accidental aspiration of instruments, and protects the soft tissues [22]. In the present study, most dental professionals showed good knowledge of hand hygiene (97.9%). According to the Center for Disease Control and Prevention (CDC), hand washing is mandatory before and after a dental procedure, before leaving the dental office, after touching any contaminated by blood, saliva or other secretions and when hands are visibly soiled [22]. Hand hygiene should be performed with soap and water for at least 20 seconds. This effectively disrupts the fatty outer layer of the virus. Alcohol-based hand rubs with 60% ethanol or 70% isopropanol are recommended as hand sanitisers [5, 23].

The present study revealed that dentists possessed a good knowledge of PPE. This is in agreement with the other studies [10, 18] and contrast to the findings of few studies [18, 24, 25]. Since the dental procedures involve face to-face communication with the patient, appropriate PPE, including masks (N-95), gloves, gowns, goggles, and face shields, are essential for infection control during COVID-19 outbreak [25]. More than half of the dentists (52.9%) displayed good practice about patient triage. Patient triage includes appropriate questionnaires on symptoms of COVID-19, recording travel history, screening for temperature, and determining the need for emergency and urgent dental care [26]. Also, telephonic screening of patients before the dental visit is recommended to identify the patients with suspected or possible COVID-19 infection [27]. In the present study, only 52.9% practiced telephonic screening before the dental visit.

Several methods have been suggested to filter the contaminated air in treatment areas such as negative-pressure room, extraoral suction, use of High-Volume Evacuator (HVE), and High-Efficiency Particulate Arrestor (HEPA) filters [28]. In the present study, 31.5 % of dentists used HEPA filters. Zhao *et al*. [29] measured the size-dependent filtration efficiency of two types of filters: the fine filters (F6 class) and High-Efficiency Particulate Air Filters and found that the HEPA filters as much as 83% of such aerosols. Therefore, for dental treatments that generate many aerosols, air purifiers with HEPA may be more effective and protective than air purifiers with fine filters [28, 29].

It was encouraging to note that the majority of the dental professionals (63.8%) in the present study showed a good attitude. All the participants agreed that it is essential to create public awareness of COVID-19 appropriate attitude and practices. Ge *et al*. [7] suggested few waiting area modifications such as displaying educational posters on COVID-19, cough etiquette instructions, provision for social distancing, and mandatory use of masks in the waiting area to prevent the spread of COVID-19. The majority of the dentists in the present study have re-organised the waiting area such that there is minimum clutter and provision for social distancing.

This study was not without any limitations. Participants were enrolled using online platforms, which could have reduced the number of responses as few might have ignored the request or would not have checked the online media. However, this method was employed to limit physical contact during the pandemic. Also, there was heterogeneity in terms of participants' original city and practising city. Hence the results of this study cannot be generalised to entire India. Again, the data presented by the participants in this study are self-reported, which might not necessarily signify actual practice. Irrespective of these limitations, our findings provide valuable information about the dentists' knowledge, attitude, and practice of infection control measures during the COVID-19 pandemic.

## Conclusion

In the present study, most of the dentists have adequate knowledge, attitude, and practice on infection control protocols in COVID-19. The study used statistical inference methods and logistic regression methods to explore and establish associations between demographic characteristics and the KAP scores obtained from the participants. The study concludes that males have a lower likelihood of good knowledge when compared with female dental professionals. However, this phenomenon sees a reversal in the case of attitude-related aspects. The study also highlights the relevance of experience in the context of its positive association with COVID-19 appropriate practices. The study has identified knowledge gaps about the usage of rubber dam, four-handed dentistry, ultrasonic instruments, and telephonic screening. This can be bridged by conducting various training programs on infection control protocols among dentists.

### What is known about this topic

- The dental aerosols can travel up to 6 feet and can harbour pathogenic microorganisms;
- Dental care providers are at increased risk of contracting COVID-19;
- The entire dental care team should be aware of the infection control protocol to prevent the spread of COVID-19.

### What this study adds

- The present study highlights the importance of telephonic screening, the role of Personal Protective Equipment, rubber dam, and hand hygiene measures during the COVID-19 pandemic;
- Our findings suggest a knowledge gap in terms of the usage of rubber dam and telephonic screening, and this calls for a large-scale awareness programme to educate the dentists regarding specific infection control protocols during the COVID-19 pandemic;
- Gender, higher qualification and years of experience of the dentists had significant association towards infection control practices during COVID-19.
